# Unmet need in the hyperlipidaemia population with high risk of cardiovascular disease: a targeted literature review of observational studies

**DOI:** 10.1186/s12872-016-0241-3

**Published:** 2016-04-26

**Authors:** S. Mitchell, S. Roso, M. Samuel, M. Pladevall-Vila

**Affiliations:** RTI Health Solutions, The Pavilion, Towers Business Park, Wilmslow Road, Didsbury Manchester, M20 2LS UK; Pfizer Ltd., Walton Oaks, Dorking Road, Walton-on-the-Hill, Tadworth, Surrey KT20 7NS UK; NUS Yong Loo Lin School of Medicine, NUHS Tower Block, Level 11, 1E Kent Ridge Road, Singapore, 119228 Singapore; RTI Health Solutions, Trav. Gracia 56 Atico 1 08006, Barcelona, Spain; The Center for Health Policy and Health Services Research, Henry Ford Health System, Detroit, MI USA

**Keywords:** Hyperlipidaemia, LDL-C, Cardiovascular, Mortality, Review, Observational studies

## Abstract

**Background:**

The aim of this study was to examine recommended target levels of low-density lipoprotein cholesterol (LDL-C) for hyperlipidaemia patients at high risk (i.e., with two or more risk factors or coronary heart disease or its risk equivalents) for cardiovascular disease (CVD); to determine LDL-C targets recommended by guidelines, and to examine the proportions of patients who do not achieve targeted LDL-C levels in real-world studies.

**Methods:**

Electronic databases were searched: Medline, Medline In-Process, Embase, BIOSIS, and the Cochrane Library (1 January 2005 to 31 December 2013). Guideline searches were limited to publications in the last 5 years. There were no geographical or language restrictions.

**Results:**

Seventeen guidelines and 42 observational studies that reported on high-risk hyperlipidaemia patients were identified. The National Cholesterol Education Program–Adult Treatment Panel III’s LDL-C target levels were the most common guidelines used for patients with very high hyperlipidaemia. However, between 68 and 96 % of patients in the studies did not achieve an LDL-C goal <70 mg/dL, except in one study conducted in China (16.9 %). In high-risk patients, 61.8 to 93.8 % did not achieve a target of <100 mg/dL. Regarding common comorbidities, patients with concomitant CVD or diabetes were least likely to reach their target LDL-C goals.

**Conclusion:**

In patients with high risk for CVD, the majority of patients do not attain recommended LDL-C goals, highlighting worldwide suboptimal hyperlipidaemia management and missed opportunities for reduction of the patients CVD risk. Lipid-modifying management strategies need to be intensified.

**Electronic supplementary material:**

The online version of this article (doi:10.1186/s12872-016-0241-3) contains supplementary material, which is available to authorized users.

## Background

Hyperlipidaemia is an increase in serum levels of one or more of the following: low-density lipoprotein cholesterol (LDL-C), total cholesterol, triglycerides, or both total cholesterol and triglycerides (combined hyperlipidaemia). Patients with hyperlipidaemia are mostly asymptomatic; however, these patients have an increased risk for cardiovascular disease (CVD), which is the main cause of premature death, and has been a major cause of disability and ill health in recent years [1–4].

Lipid-lowering medications include statins, fibrates, and anion-exchange resins; they are recommended as part of the management strategy for primary or secondary prevention of CVD in adults with a 20 % or greater 10-year risk of developing CVD [5]. In large, randomised, controlled trials, statins in particular have been shown to be effective in preventing coronary heart disease events and in reducing overall mortality [4, 6–8]. Statins therefore are recommended as first-line therapy, whereas fibrates and anion-exchange resins are considered second-line therapy, or combination therapy when used with statins [9]. Guidelines published for Europe [4] and other countries before 2013 [10, 11] recommend a treat-to-goal paradigm for LDL-C levels. However, despite clear evidence that there is a positive association between LDL-C levels and the risk of cardiovascular (CV) events, there is inconclusive evidence that achieving specific goal levels reduces the risk of CV events.

A number of published studies have reported that patients do not achieve LDL-C goals in the hyperlipidaemia population [12, 13]; however, to our knowledge the latest evidence has not been collated in a rigorous manner. The current study performed a targeted review of the published guidelines to identify the recommended treatment targets for LDL-C levels in clinical practice. This review also assembled the best evidence from recently published observational studies to determine the proportions of very high-risk, high-risk, and moderately high-risk patients who do not achieve targeted LDL-C levels in a real-world setting—that is, in routine clinical practice—and, if reported in studies, the reasons for not achieving target levels. This review provides a qualitative overview of the available data; a meta-analysis was not performed and a statistical analysis of the results was not undertaken.

## Methods

### Literature search and data extraction

This review was performed in an unbiased manner by using a prespecified protocol and an explicit, reproducible plan for the literature search and synthesis. A targeted literature search to identify observational studies was performed in the following databases: Medline, Medline In-Process, Embase, BIOSIS, and the Cochrane Library (from 1 January 2005 to 31 December 2013). Guideline searches of these databases were limited to publications in the last 5 years in order to evaluate the most recent practice patterns and recommendations. Hand searches also were performed, including a search of the Agency for Health Care Research and Quality’s National Guideline Clearing House. Systematic reviews were used to identify primary studies but were not included in this review. No limitations on publication language or geographic perspective were applied. Articles that were published in a non-English language were translated as required.

Search terms to identify guidelines and other primary studies included combinations of free text and Medical Subjects Headings (MeSH) and consisted of the following sets of terms:

- ▪ Health condition of interest (e.g., “Hypercholesterolemia”[Majr], hypercholesterol*[Title], Hyperlipidemias[Majr], hyperlipidemia[Title], hyperlipidemias[Title], hyperlipidaemia[Title], hyperlipidaemias[Title])
- ▪ Suboptimal response (e.g., sub-optim*[Title/Abstract], suboptim*[Title/Abstract], sub optim*[Title/Abstract], “goal”[Title/Abstract], “target”[Title/Abstract], optimum[Title/Abstract], achieve*[Title/Abstract])
- ▪ Outcomes of interest (e.g., LDL-C goal)
- ▪ Guidelines and clinical studies (“Practice Guidelines as Topic”[MeSH], “Guidelines as Topic”[MeSH], “Practice Guideline”[Publication Type], “Prospective Studies”[MeSH], “Registries”[MeSH], observational stud*[Text Word], “Retrospective Studies”[Majr]).

The full Medline literature search strategy is presented in Additional file 1: Table S1; this search strategy was adapted for other databases.

Screenings of titles, abstracts, and full-text articles for eligibility were performed by one researcher; a second researcher performed a quality check of a random selection of 10 % of all references identified from the searches; any disagreement was resolved by consensus, with input from an experienced senior researcher. The following were the predefined inclusion criteria:

- *Population*: Adult patients with hyperlipidaemia with moderately high risk, high risk, or very high risk of CVD
- *Intervention:* No limits applied
- *Outcomes*: Target LDL-C levels recommended by guidelines; the proportion of patients who did not achieve the LDL-C targets; and the reasons, if reported in the study, for not achieving the target LDL-C levels
- *Study type*: Observational studies; clinical practice guidelines
- *Exclusions*: Studies with a sample size of less than 100 subjects or studies in familial hypercholesterolaemia

### Data synthesis

The treatment guidelines obtained from various countries were used to examine differences among recommended LDL-C target levels for patients with hyperlipidaemia. Results of the observational studies generally were summarised quantitatively, using descriptive statistics; due to the heterogeneity between the included studies a statistical analysis was not undertaken.

To qualitatively synthesise data from the observational studies, we summarised data in detailed evidence tables and figures. The summarised data included information on the study design; population size; patient characteristics, including risk category definitions (very high risk, high risk, and moderately high risk of CVD); and results (i.e., the LDL-C target level recommended for hyperlipidaemic patients who are at very high risk, high risk, and moderately high risk of CVD; the proportion of patients who do not achieve the LDL-C target levels; and the reasons for not achieving these targets). The definition of high risk was extracted as reported by the study; generally high risk was defined as two or more risk factors for coronary heart disease (CHD) or its risk equivalents. A quality assessment of the included studies was not performed because this review did not evaluate comparative effectiveness and safety of treatments used in the management of hyperlipidaemia; rather, we evaluated, within the studies reviewed, the general trends in patients not achieving LDL-C target goals.

## Results

### Study identification and characteristics

**Fig. 1:**
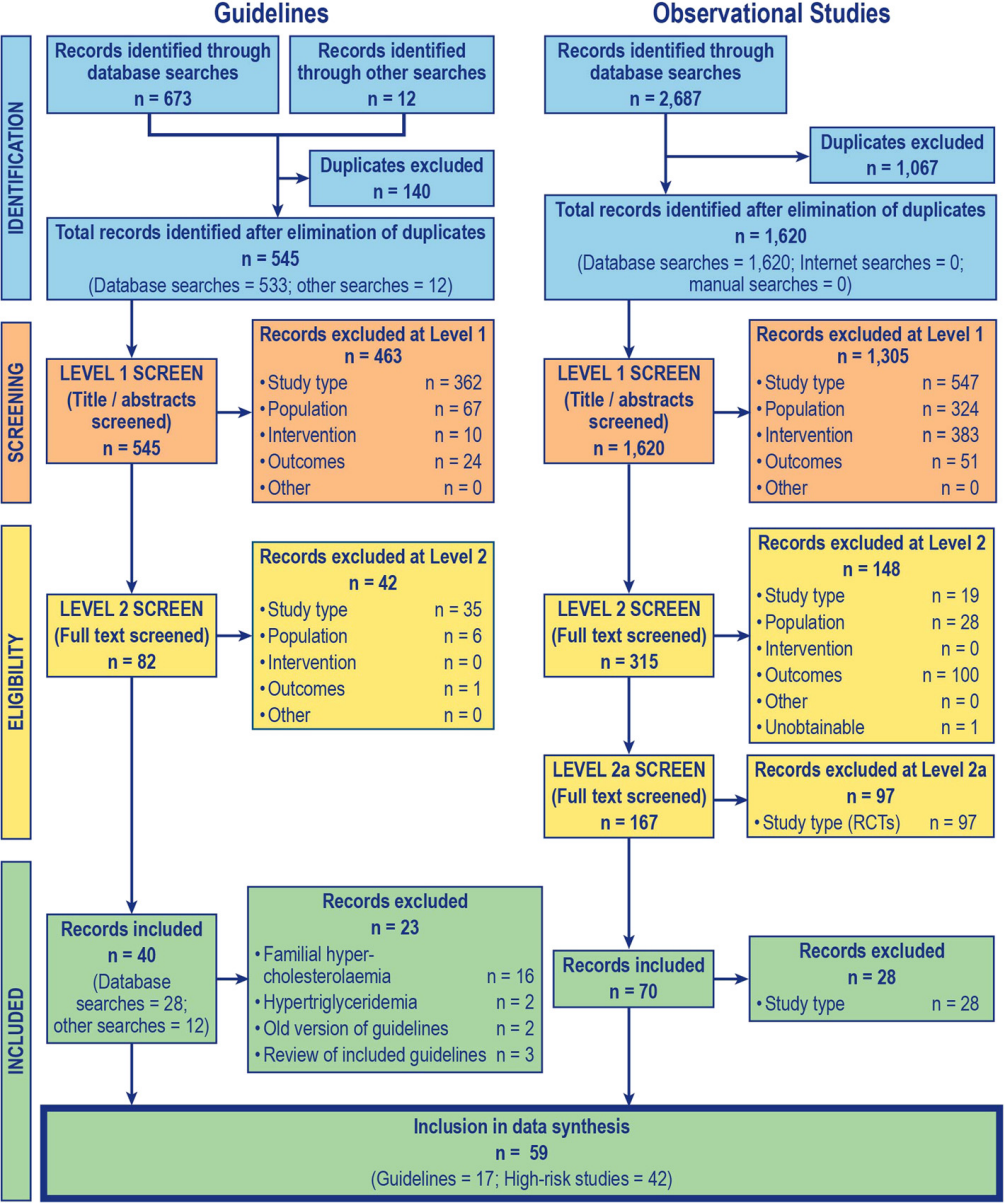
Flow Chart for Study Inclusion and Exclusion. *PRISMA* preferred reporting items for systematic reviews and meta-analyses, *RCT* randomised, controlled trial

Searches for guidelines identified 545 records (databases = 533; Internet and hand searches = 12). After the initial screening of titles and abstracts (level 1 screening), 82 publications (databases searches = 70; Internet and hand searches = 12) were selected for full-text (level 2) screening. Ultimately, 17 publications, describing treatment guidelines from more than 20 countries, were included in the current review. Searches for clinical studies retrieved 1620 records (databases = 1620; Internet and hand searches = 0) and 42 observational studies that reported data for high-risk patients (that is, patients with very high risk, high risk, and moderately high risk of CVD) were included. The volume of studies included and excluded at each stage of screening is shown in the Preferred Reporting Items for Systematic Reviews and Meta-Analyses flow chart [14] presented in Fig. 1.

### Treatment guidelines

The European Society of Cardiology and the European Atherosclerosis Society (ESC/EAS) guidelines for LDL-C target levels for patients with hyperlipidaemia are widely used in European clinical practice in Austria, Belgium, Denmark (endorsed by the Danish Society of Cardiology), France, Ireland, Italy (Nota 13, the prescribing guideline for hyperlipidaemia, references the ESC/EAS), Poland (recommended by Polish Cardiology Association), and Russia. Additionally in Austria, there is a consensus document, *Lipidkonsensus*, prepared by experts from eight Austrian medical associations [15, 16]. In Finland, the Netherlands, Norway, Spain, Sweden, Switzerland, the United Kingdom, and South Africa, experts have developed, or are planning to develop, country-specific guidelines; the recommended LDL-C target levels for various risk categories in these guidelines varied slightly compared with the levels recommended in ESC/EAS guidelines (Additional file 1: Table S2). The new guidelines from the American College of Cardiology and the American Heart Association (ACC/AHA) developed in conjunction with the National Heart, Lung, and Blood Institute do not recommend a target LDL-C level for treated patients because, according to the authors, there is a lack of evidence from clinical trials. Rather, the ACC/AHA guidelines recommend intensive treatment options based on risk assessment and LDL-C levels to reduce CVD events. In Asia, most countries currently follow the National Cholesterol Education Program–Adult Treatment Panel III (NCEP-ATP-III) guidelines.

#### Recommended LDL-C target levels for patients with varying underlying risks

##### Recommended LDL-C target levels for very high-risk or high-risk patients

This review observed that the risk definitions were not consistently used between guidelines. Eleven guidelines recommended target LDL-C levels for patients with a high or very high risk for CV disease (Additional file 1: Table S2). For patients with CHD, or who have two or more CVD risk factors, CHD risk-equivalent conditions, or diabetes, the recommended LDL-C target levels ranged from <70 mg/dL (<1.8 mmol/L) to <120 mg/dL (3.1 mmol/L); the majority of the guidelines recommended a target of <100 mg/dL (<2.6 mmol/L) [11, 15–21]. Two guidelines from Europe [5, 20] recommended a target LDL-C level <70 mg/dL (<1.8 mmol/L) for very high-risk patients; the ACC/AHA guidelines [1] recommended treatment with a moderate- or high-intensity statin (depending on the patient’s 10-year atherosclerotic CVD risk) in patients with a LDL-C level of between 70 and 189 mg/dL (1.8–4.9 mmol/L).

Patients with known CVD are at very high risk for CV events. Long-term treatment to prevent recurrent cardiac morbidity and mortality and to improve quality of life in patients who had a prior episode of myocardial infarction, acute coronary syndrome, angina, stroke, peripheral artery disease, or peripheral vascular disease, or who are at high risk of ischaemic cardiac events for other reasons, such as severe coronary artery stenoses or prior coronary surgical procedures [22], is discussed as secondary prevention. Thirteen treatment guidelines recommended specific LDL-C targets for secondary prevention. The most common target was <100 mg/dL (<2.6 mmol/L), which was recommended by eight different guidelines in Asia [17]. For very high-risk patients, Philippines and Thailand recommend a lower target level of less than 70 mg/dL (1.8 mmol/L). In the United Kingdom, the guidelines published by the National Collaborating Centre for Primary Care and Royal College of General Practitioners [5] set a target LDL-C level of ≤77 mg/dL (≤2.0 mmol/L) for adults with clinical evidence of CVD. A broader target of between 66 and 97 mg/dL (1.7 and 2.5 mmol/L) is recommended by the Norwegian Directorate of Health, for persons with atherosclerosis or CVD.

##### Recommended LDL-C target levels for moderately high-risk patients

Eleven guidelines recommended target LDL-C levels for moderately high-risk patients (those with no CHD but with ≥2 risk factors) (Additional file 1: Table S2). The recommended targets ranged from 77 mg/dL (≤2.0 mmol/L) in the Canadian Cardiovascular Society guidelines [23] to <140 mg/dL (3.6 mmol/L) in the Japanese Atherosclerosis Society guidelines [11]. Most guidelines recommended a target of <130 mg/dL (3.4 mmol/L) [15–19, 24].

### Proportion of high- or very high-risk patients not achieving LDL-C targets

**Fig. 2:**
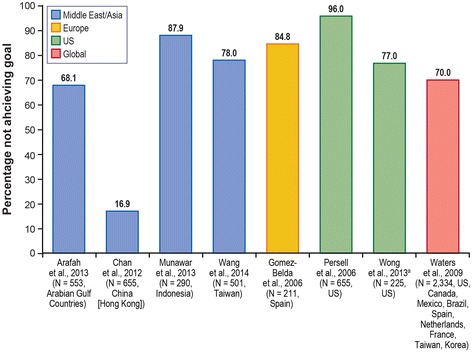
Patients (Very High Risk) Not Achieving NCEP-ATP-III Guidelines LDL-C Level Target, <70 mg/dL (1.81 mmol/L). *LDL-C* low-density lipoprotein cholesterol, *NCEP-ATP-III* National Cholesterol Education Program–Adult Treatment Panel III, *US* United States. Notes: Treated and untreated patients. The N represents patients at high risk, a subset of the total number of patients studied. Sources: [29, 35–37, 39, 43–45]

**Fig. 3:**
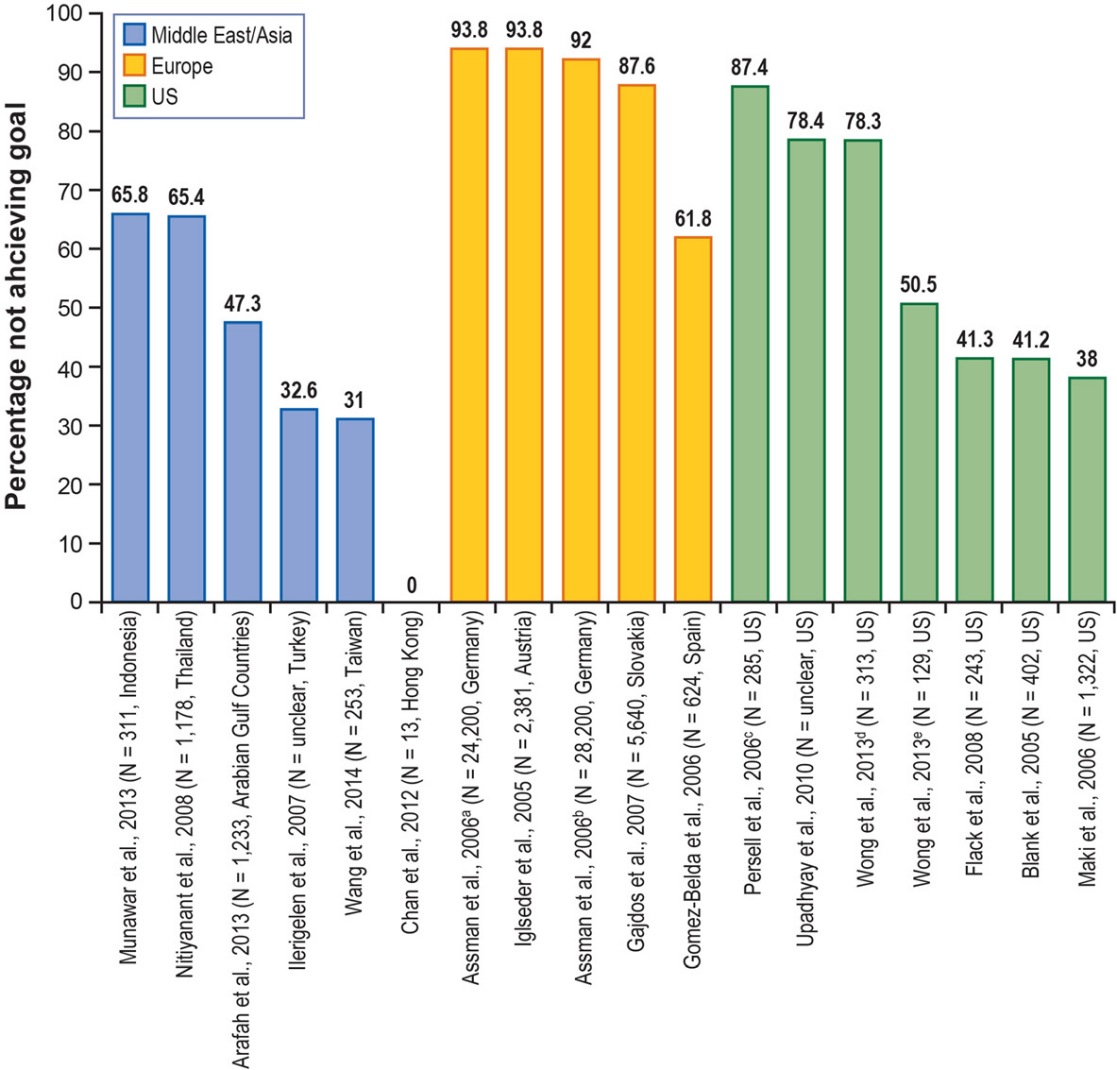
Patients (High Risk) Not Achieving NCEP-ATP-III Guidelines LDL-C Level Target, < 100 mg/dL (2.56 mmol/L). *LDL-C* low-density lipoprotein cholesterol, *NCEP-ATP-III* National Cholesterol Education Program–Adult Treatment Panel III, *US* United States. ^a^ Primary prevention, 9 months, women. ^b^ Primary prevention, 9 months, men. ^c^ Based on 2004 guidelines. ^d^ Treated and untreated patients. ^e^ Treated patients. Sources: [25, 26, 29, 34–36, 39, 43–51]

**Fig. 4:**
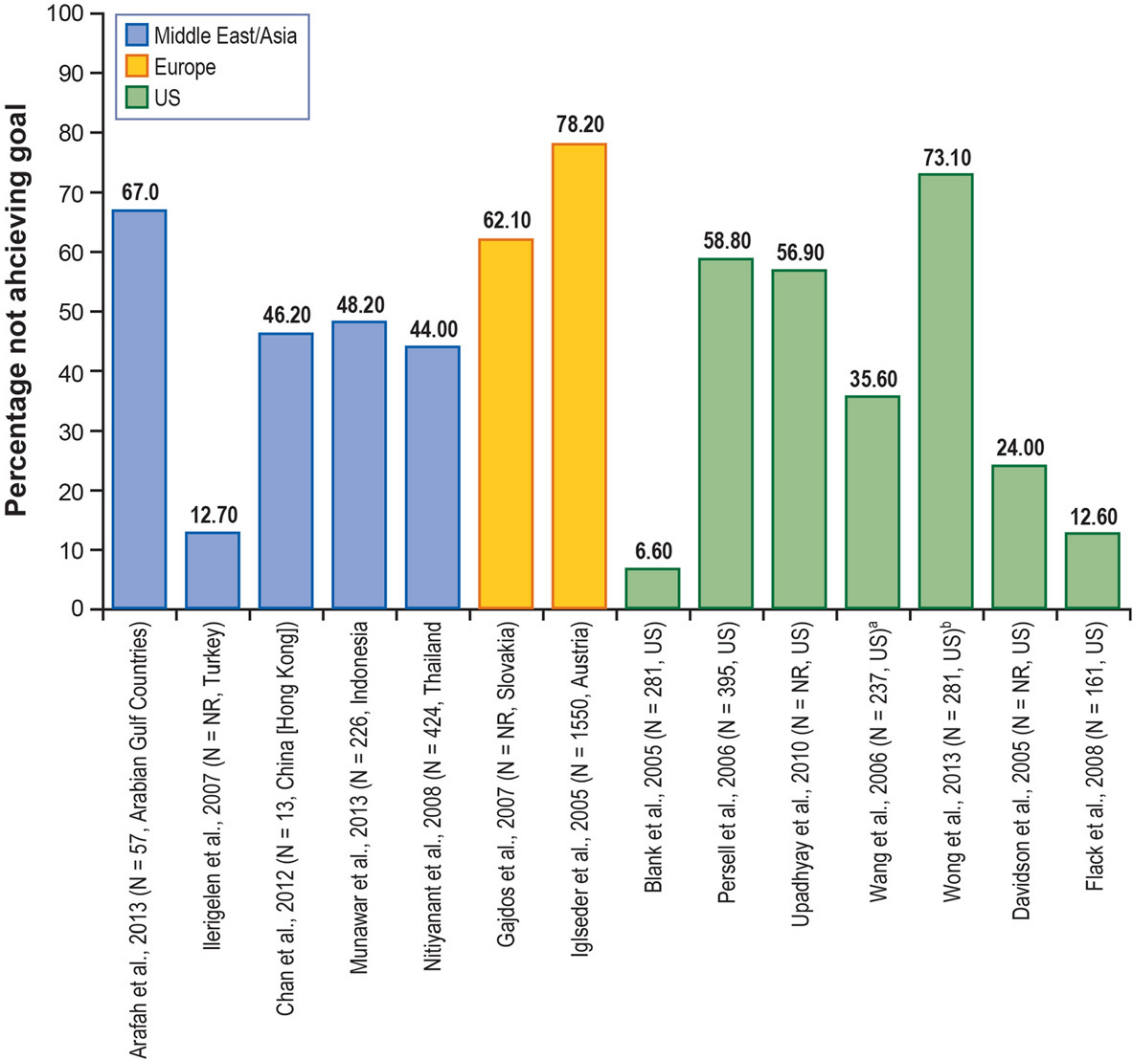
Patients (Moderately High Risk) Not Achieving NCEP-ATP-III Guidelines LDL-C Level Target, < 130 mg/dL (3.0 mmol/L). *LDL-C* low-density lipoprotein cholesterol, *NCEP-ATP-III* National Cholesterol Education Program–Adult Treatment Panel III, *NR* not reported, *US* United States. Note: The N represents patients at high risk, a subset of the total number of patients studied. ^a^ Based on data from 2003. ^b^ Treated and untreated patients. Sources: [25, 29, 35, 36, 44–53]

Numerous clinical and epidemiologic studies have shown that an elevated LDL-C level is one of the major modifiable risk factors associated with the development of CHD. Of the observational studies that were included in the current review, NCEP-ATP-III LDL-C targets were most commonly used, followed by the Canadian Working Group, and the Third Joint European Task Force of the European Society of Cardiology; other studies used country-specific guidelines. In very high-risk patients, between 68 and 96 % did not achieve an LDL-C goal of <70 mg/dL, as recommended by the NCEP-ATP-III guidelines (Fig. 2), with the exception of one study conducted in China (16.9 %). Most studies found that most high-risk patients (61.8–93.8 %) did not achieve a target of <100 mg/dL as recommended by the NCEP-ATP-III guidelines (Fig. 3); nine studies reported lower proportions (0.0–47.3 %). For moderately high-risk patients (Fig. 4) in 10 out of 14 studies, 35.6 to 78.2 % did not achieve the NCEP-ATP-III target goal of <130 mg/dL. Further, four studies conducted in the United States reported lower proportions, between 6.6 and 24.0 %. Similar results were observed in Ilerigelen et al. [25] (12.7 %) which was conducted in Turkey.

Three studies that followed the Canadian Working Group guidelines, using a target of <97 mg/dL (2.5 mmol/L), reported that between 38.0 and 42.3 % of high-risk patients did not achieve this goal [26–28]. A further five studies also used a target of <97 mg/dL (<2.5 mmol/L), as recommended by the Third Joint European Task Force guidelines, the European Society of Cardiology, and guidelines from Brazil and Hungary; 41.7 to 89.7 % patients did not achieve this goal [29–33].

### Proportion of high- or very high-risk patients with concomitant conditions not achieving LDL-C targets

Overall, of all of the common comorbidities, patients with concomitant CVD and diabetes seemed the least likely to reach their target LDL-C goals (Additional file 1: Table S3).

Concomitant health conditions may have an impact on hyperlipidaemia patients’ CV risk category and, by extension, their LDL-C target. This is particularly true for CHD and other conditions that are classed as CHD risk equivalents because these are considered to be the highest risk indicators for CVD, and therefore have the most stringent LDL-C targets. The studies we reviewed showed considerable variability in LDL-C goal achievement for patients with different concomitant conditions (Additional file 1: Table S3). In the majority of the studies, more than 60 % of patients failed to achieve the LDL-C target levels. In a 4E-Registry study [34], the percentage of male patients with diabetes mellitus failing to achieve goal levels was similar to the percentage of male patients without diabetes failing to achieve goal levels (74.7 % vs. 71.7 %). Women without diabetes mellitus, however, did much better in achieving their individual lipid goals: 55 % did not attain the goal levels, compared with 76 % of women with diabetes. However, achievement of treatment goals in patients with diabetes was just as poor as in other high-risk groups in the 4E cohort. In a cross-sectional survey conducted in Asia [35], >70 % of patients with diabetes, CHD, carotid artery disease, peripheral arterial disease, metabolic syndrome, or other multiple risk factors (10-year CHD risk >20 %) did not achieve the recommended LDL-C targets.

Some studies observed that the number of patients with CHD who do not achieve their LDL-C targets was not as high (<50 %); but most studies reported that the majority of patients did not meet their targets. A similar trend was observed in patients with other concomitant conditions such as hypertension, stroke, and kidney disease. One common theme among the studies that compared concomitant conditions was that patients with multiple conditions (i.e., patients at higher CV risk) were less likely to reach LDL-C goals than patients with a single condition. Another notable theme was that, within an individual study, patients in higher CV risk categories were less likely to reach LDL-C target goals than patients in lower CV risk categories. However, it should be noted that patients in higher risk categories have lower target goal levels.

#### Potential reasons for patients not achieving target LDL-C levels

The reasons why patients did not achieve their LDL-C targets was not consistently investigated nor reported across the studies included in this review. Of the studies that did report reasons, only a few conducted univariate or multivariate analysis to investigate the relationship between patient-level factors that may have contributed to failure to reach targets. Patient characteristics such as gender; age; race; body mass index; and comorbid conditions such as diabetes, CHD, peripheral arterial disease, and carotid arterial disease are some of the known risk factors that influence the achievement of LDL-C target goals. However, it is difficult to reach any firm conclusions because some of the studies report conflicting evidence.

Two studies, one of which was a large international survey, reported that males were more likely to attain their LDL-C target goals than females [36, 37], with a univariate analysis finding that being female decreased the odds of attaining the LDL-C goal (odds ratio = 0.62, 95 % CI, 0.39-0.99; *P* = 0.043) [36]. Contrary to these findings, two large studies reported that successful LDL-C goal attainment was positively associated with being female [29, 38].

A Chinese study found that older age increased the odds (odds ratio = 1.02; 95 % CI, 1.00–1.05 for every 10 years increment in age; *P* = 0.038) of attaining the LDL-C target goals [36]. Older age was also found to be a multivariate predictor of successful LDL-C goal achievement in a worldwide multicenter study [37]. Conversely, a large study in the US found that older age was negatively associated with LDL-C control [38]. Similarly, another study reported that LDL-C goal attainment was significantly related to an age <40 years [29].

As might be expected, several studies reported that baseline total cholesterol or LDL-C levels had an inverse relationship with LDL-C target achievement, i.e., higher baseline total cholesterol or LDL-C was associated with significantly lower odds of attaining LDL-C goals, *P* <0.001 [35, 36, 39]. Baseline CV risk was also found to influence attainment of LDL-C goals. Two studies conducted in Asia [35, 39] suggested that goal attainment was inversely related to baseline CV risk, i.e., the higher the CV risk at baseline, the less likely the patients were to reach their target. These findings were supported by a large multicentre, international trial, which found that being in a lower risk CV group was a multivariate predictor of successful LDL-C goal achievement [37].

Patient adherence has been considered to be a major factor in the low rate of LDL-C target attainment; higher adherence to treatment is linked to higher proportions of patients reaching their targets [35]. Reasons for non-adherence were cited as patients forgetting to take their medication or stopping taking their medication when their cholesterol had returned to normal [33, 35, 40]. A cross-sectional study in Brazil suggested that non-adherence was common in the population with scarce financial resources due to the high cost of medication [30]. Improper communication between health care professionals and the patients, which was particularly clear among aged patients or those with poor literacy, was also found to be an important factor for non-adherence [30].

Whilst current guidelines for treating hyperlipidaemia provide specific algorithms for the treatment of chronic conditions, they also present a more complex approach to lipid management, which requires physicians to make decisions about multiple options for treating each patient. A cross-sectional survey conducted in the United States [41] showed that although a significant minority of patients did not receive a dose increase when one appeared warranted, approximately 70 % of patients did receive a dose increase at some point. There was a highly significant association of several physician attitudes and beliefs with the decision to increase the statin dose; physicians who believed that “statins are effective” were more likely to increase statin dose, whereas those who had an attitude that “close enough to goal is good enough” were less likely to switch or titrate to higher statin dose [41]. Of the patients who received an increase in statin dose, 50 % were still not at goal and few were on the highest doses, suggesting that physicians are not “treating to goal” [41].

## Discussion

In this review, evidence from the observational studies shows that despite the increasing global awareness of the need for the management of CV events, more than half of high-risk patients do not attain the lipid levels recommended by the published guidelines. Although lipid-lowering drugs such as statins are proven to have beneficial effects on long-term outcomes, hyperlipidaemia remains unsatisfactorily managed in routine clinical practice. The ESC/EAS and NCEP-ATP-III guidelines are the primary guidelines used in clinical practice around the world; they recommend a treat-to-goal paradigm. In the United States, the ACC/AHA’s latest guidelines [1] do not recommend a target LDL-C level; rather, they recommend intensive treatment options based on risk assessment and LDL-C levels. The panel believes that the use of LDL-C targets may result in the overtreatment of patients with non-statin drugs that have not shown to reduce the risk of CV events [1].

Some studies suggested that achieving LDL-C goals was inversely related to baseline CV risk [35, 37, 39], although one of the reasons put forward for not achieving goals is that the target levels are lower for such patients (<70 mg/dL). Treatment non-adherence also is cited as a common issue in various studies [27, 30, 33, 35]. It is thought that non-adherence may be due to scarce financial resources [30] or to improper communication between the health care professional and the patient, particularly among elderly patients and those with poor literacy. In addition, poor physician adherence to treatment guidelines could also play a crucial role. Other reasons for poor attainment of treatment goals can include inadequate dosing, failure to properly up titrate the dose, not switching to a more potent drug when necessary, and a lack of follow-up after initiation of treatment. According to a 4E Registry study in Germany, treatment, once started, was rarely modified. The statin dose was increased in only 10 % of patients; leading to the assumption that doctors are not aware of how to reach target values [34]. In observational studies, patients and their physicians select treatment on the basis of clinical need or preference, which can result in differences in clinical outcomes solely because of differences between those who receive and those who do not receive a treatment. These results must be interpreted in consideration of the relatively high rate of missing data at the follow-up visits. It is possible that predominantly ‘difficult-to-treat’ or non-adherent patients were lost. Good management strategies, appropriate therapeutic approaches, and good patient and physician adherence to recognised practice guidelines will be crucial in achieving favourable outcomes. Patients in a high-risk or a very high-risk category tend to have lower achievement of LDL-C goals, highlighting suboptimal hyperlipidaemia management worldwide or even the setting of unrealistic goals [42]. Patients in higher CV risk categories tend to have more stringent LDL-C target levels, which may contribute to failure to achieve target levels. Limited evidence suggests that the reasons for not achieving target LDL-C goals include gender, age, comorbidities (e.g., diabetes and CV risk), hypertension, baseline LDL-C and total cholesterol levels, and choice of treatments and dosages. Further primary studies are needed to rigorously explore these reasons.

## Conclusion

The results of the current review suggest there are several unmet needs in treating hyperlipidaemic patients: the reduction of the patients’ risks for CVD and the consequent reduction of the occurrence of CV events have confirmed the necessity of intensifying lipid-modifying management. The failure of large numbers of patients to achieve LDL-C targets, and specifically the failure of patients in high-risk or very high-risk categories, to attain LDL-C goals in a number of countries highlight suboptimal hyperlipidaemia management worldwide.

## Additional file

Additional file 1:
**Table S1**. MEDLINE Literature Search Strategy. **Table S2**. Recommended LDL-C Targets for High-Risk Patients From Treatment Guidelines. **Table S3**. Patients With Pre-existing Conditions Not Achieving Target LDL-C Levels. (DOCX 63 kb)
