# Supplementary material for: Unmet need in the hyperlipidaemia population with high risk of cardiovascular disease: a targeted literature review of observational studies
Source: BMC Cardiovasc Disord. 2016 Apr 26;16:74. doi: 10.1186/s12872-016-0241-3 (PMC4845323; doi:10.1186/s12872-016-0241-3)
Supplement: Additional file 1: — Table S1. MEDLINE Literature Search Strategy. Table S2. Recommended LDL-C Targets for High-Risk Patients From Treatment Guidelines. Table S3. Patients With Pre-existing Conditions Not Achieving Target LDL-C Levels. (DOCX 63 kb) [file 12872_2016_241_MOESM1_ESM.docx]

SUPPLEMENTARY MATERIAL

Table S1. MEDLINE Literature Search Strategy

| Term Group | Search Number | Search Terms | Hits |
| --- | --- | --- | --- |
| Population of interest | 1 | “Hypercholesterolemia”[Majr] OR hypercholesterol*[Title] OR Hyperlipidemias[Majr] OR hyperlipidemia[Title] OR hyperlipidemias[Title]  OR hyperlipidaemia[Title] OR hyperlipidaemias[Title] | 37,863 |
| Clinical studies (observational) | 2 | “Clinical Trial, Phase IV”[Publication Type] OR “Cohort Studies”[MeSH] OR cohort*[Text Word] OR “longitudinal”[Text Word] OR “Longitudinal Studies”[MeSH] OR “Follow-Up Studies”[MeSH] OR evaluation stud*[Text Word] OR “Prospective Studies”[MeSH] OR “Registries”[MeSH] OR observational stud*[Text Word] OR “Case-Control Studies”[Majr] OR “Retrospective Studies”[Majr] OR “Cross-Sectional Studies”[Majr] OR (“Clinical Trials as Topic”[MeSH:NoExp] AND “Follow-Up Studies”[MeSH]) OR (“Follow-Up Studies”[MeSH] AND (open-label*[Text word] OR open-label stud* OR non-blinded stud*[Text word])) | 1,789,560 |
| Clinical studies (RCTs) | 3 | “Randomized Controlled Trials as Topic”[MeSH] OR randomized controlled trial*[Text Word] OR randomised controlled trial*[Text Word] OR randomized clinical trial*[Text Word] OR randomised clinical trial*[Text Word] OR randomized trial*[Text Word] OR randomised trial*[Text Word] OR “randomly”[Title/Abstract] OR “random allocation”[Text Word] OR allocated random*[Text Word] OR random assignment*[Text Word] OR “Clinical Trial, Phase II”[Publication Type] OR “Clinical Trial, Phase III”[Publication Type] | 681,770 |
| Suboptimal response | 4 | sub-optim*[Title/Abstract] OR suboptim*[Title/Abstract] OR sub optim*[Title/Abstract] OR “goal”[Title/Abstract] OR “target”[Title/Abstract] OR optimum[Title/Abstract] OR achieve*[Title/Abstract] | 1,198,432 |
|  | 5 | #2 OR #3 OR #4 | 3,315,022 |
| Outcomes | 6 | (LDL-C AND (goal* OR achieve* OR target*)) OR LDL-C OR “low density lipoprotein” OR “low density lipoproteins” OR “Cholesterol, LDL” | 67,451 |
|  | 7 | #1 AND #5 AND #6 | 4,435 |
| Exclusion terms—exclude animals | 8 | “Animals”[MeSH] NOT “Humans”[MeSH] | 3,844,728 |
| Exclusion terms—study type | 9 | “Comment”[Publication Type] OR “Letter”[Publication Type] OR “Editorial”[Publication Type] OR “Case Reports”[Publication Type] OR “Clinical Trial, Phase I”[Publication Type] | 2,780,501 |
| All relevant studies | 10 | #7 NOT (#8 OR #9) | 4,135 |
| Limits | 11 | Publication date: 2005 to present | 1,737 |
|  | 12 | (Adult[MeSH] OR adults OR adult) AND #11 | **1,290** |

MeSH = Medical Subject Heading; RCT = randomised, controlled trial.

Table S2. Recommended LDL-C Targets for High-Risk Patients From Treatment Guidelines

| LDL-C Target Level | Patient Population/Risk Category | Organisation  Reference(s) | Country |
| --- | --- | --- | --- |
| **Very high risk** | | | |
| < 70 mg/dL (< 1.8 mmol/L) | Very high risk: acute coronary syndrome; stable CHD and T2DM; stable CHD and metabolic syndrome; peripheral arterial occlusive disease; progressive or recurrent CHD despite LDL-C < 100 mg/dL | Austrian Diabetes Association  Wascher et al. (2012)[1] | Austria |
| < 70 mg/dL (< 1.8 mmol/L) and/or ≥ 50% reduction when target level not reached | Very high CV risk (established CVD, T2DM, T1DM with target organ damage, moderate to severe chronic kidney disease, or a SCORE level ≥ 10%) | ESC/EAS  Reiner et al. (2011)[2] | Europe |
| < 70 mg/dL (< 1.8 mmol/L) | Very high risk | NCEP-ATP-III guidelines  Grundy et al. (2004)[3]; Hankey et al. (2010)[4] | US, China (follows NCEP-ATP-III) |
| **High risk** | | | |
| < 120 mg/dL (3.1 mmol/L) | Category 3, high risk (10-year risk of death from CVD ≥ 2%) | Japanese Atherosclerosis Society  Teramoto et al. (2013)[5] | Japan |
| < 100 mg/dL (2.6 mmol/L) | High risk: > 2 risk factors, SCORE 10-year risk ≥ 5%, FRS 10-year risk > 20%, CHD, cerebrovascular disease, T2DM, T1DM and aged > 40 years, or nephropathy | Austrian Lipid Consensus *Lipidkonsensus*  Austrian Lipid Consensus (2010)[6]; Huber et al. (2011)[7] | Austria |
|  | High risk: CHD or CHD risk equivalents or diabetes | Korean Stroke Society  Hankey et al. (2010)[4] | Korea |
|  |  | Malaysian Society of Neurosciences  Hankey et al. (2010)[4] | Malaysia |
|  |  | Swiss Atherosclerosis Association  Rodondi et al. (2011)[8] | Switzerland |
| < 100 mg/dL (2.6 mmol/L) | High risk: CHD^a^ or CHD risk equivalents^b^ (10-year risk > 20%) | NCEP-ATP-III guidelines  Grundy et al. (2004)[3]; Hankey et al. (2010)[4] | US, China (follows NCEP-ATP-III) |
| < 97 mg/dL (< 2.5 mmol/L) | High CV risk (markedly elevated single risk factors, a SCORE level ≥ 5 to < 10%) | ESC/EAS  Reiner et al. (2011)[2] | Europe |
|  | CVD, T2DM or T1DM with microalbuminuria, severe genetic lipid disorders (e.g., familial hypercholesterolaemia), or persistent asymptomatic CHD risk (> 20%) despite lifestyle change | South African Medical Association and Lipid and Atherosclerosis Society of Southern Africa (2000)[9]  Butler (2010)[10] | South Africa |
| ≤ 77.6 (2.0 mmol/L) or ≥ 50% reduction | High risk (previous MI, clinical atherosclerosis, abdominal aortic aneurysm, diabetes of > 15 years’ duration and age > 30 years, diabetes and age > 40 years, microvascular disease, high-risk kidney disease, high-risk hypertension, FRS: ≥ 20% | Canadian Cardiovascular Society  Anderson et al. (2013)[11] | Canada |
| ≤ 70 mg/dL (< 1.8 mmol/L) | High risk: CHD or CHD risk equivalents (T2DM, T1DM with microalbuminuria, atherosclerosis, peripheral vascular disease), FRS 10-year risk of CHD event: > 20% | Caribbean Cardiac Society  Chung (2008)[12] | West Indies |
| **Moderately high risk** |  |  |  |
| < 140 mg/dL (3.6 mmol/L) | Intermediate risk (10-year risk of death from CVD ≥ 0.5 to < 2%, with no additional risk factors or < 0.5% with presence of 1 or more risk factors^c^) | Japanese Atherosclerosis Society  Teramoto et al. (2013)[5] | Japan |
| < 130 mg/dL (3.4 mmol/L) | No CHD and ≥ 2 risk factors | Korean Stroke Society  Hankey et al. (2010)[4] | Korea |
|  |  | Indonesian Neurological Association  Hankey et al. (2010)[4] | Indonesia |
|  | Medium risk: 2 risk factors; SCORE 10-year risk: 3%-4%, FRS 10-year risk: 10%-20% | Austrian Lipid Consensus *Lipidkonsensus*  Austrian Lipid Consensus (2010)[6]; Huber et al. (2011)[7] | Austria |
|  | Moderate risk: ≥ 2 risk factors; FRS 10-year risk: < 10% | Caribbean Cardiac Society  Chung (2008)[12] | West Indies |
|  |  | NCEP-ATP-III guidelines  Grundy et al. (2004)[3]; Hankey et al. (2010)[4] | US, China (follows NCEP-ATP-III) |
|  | Medium risk | Swiss Atherosclerosis Association  Rodondi et al. (2011)[8] | Switzerland |
| < 130 mg/dL (optional goal: < 100 mg/dL) | Moderately high risk: ≥ 2 risk factors^d^ (FRS 10-year risk: 10%-20%) | NCEP-ATP-III guidelines  Hankey et al. (2010)[4] | China |
| < 115 mg/dL (< 3.0 mmol/L) | Moderate risk (SCORE level: > 1 to ≤ 5%) | ESC/EAS  Reiner et al. (2011)[2] | Europe |
|  | Asymptomatic individuals with initial 10-year CHD risk < 20%, or for initial 10-year CHD risk > 20% but reduced to < 20% with lifestyle changes | South African Medical Association and Lipid and Atherosclerosis Society of Southern Africa (2000)[9]  Butler (2010)[10] | South Africa |
| < 100 mg/dL (< 2.6 mmol/L) | Moderately high risk: ≥ 2 risk factors; FRS 10-year risk: 10%-20% | Caribbean Cardiac Society  Chung (2008)[12] | Caribbean |
| ≤ 77mg/dl (2.0 mmol/L) or ≥ 50% reduction of LDL-C | Intermediate risk identified through screening (adjusted FRS: ≥ 10% and < 20%); treat if LDL-C ≥ 3.5 mmol/L | Canadian Cardiovascular Society  Anderson et al. (2013)[11] | Canada |
| **Secondary prevention** |  |  |  |
| < 100 mg/dL (2.6 mmol/L) (or > 30%-40% reduction [Chinese Expert Panel]) | Patients with ischaemic stroke or TIA | Working Group on Stroke and Lipid Management in Asia Consensus Panel  Hankey et al. (2010)[4] | Asia (consensus statement) |
|  |  | Ministry of Health, Singapore  Hankey et al. (2010)[4] | Singapore |
|  |  | Chinese Expert Panel  Hankey et al. (2010)[4] | China |
| < 100 mg/dL (2.6 mmol/L) (Philippines and Thailand additional target for very high-risk patients: < 70 mg/dL [1.8 mmol/L]) | Patients with CHD or symptomatic atherosclerotic disease | Indonesian Neurological Association  Hankey et al. (2010)[4] | Indonesia |
|  |  | Stroke Society of the Philippines  Hankey et al. (2010)[4] | Philippines |
|  |  | NR  Hankey et al. (2010)[4] | Thailand |
|  |  | Korean Stroke Society  Hankey et al. (2010)[4] | Korea |
|  |  | Japanese Atherosclerosis Society  Teramoto et al. (2013)[5] | Japan |
| < 2.5 mmol/L | Atherosclerotic disease | Swedish Board of Health and Welfare | Sweden |
|  | Patients with CVD or T2DM | Medical Council of the Dutch Institute for Health care Improvement  Smulders et al. (2008)[13] | Netherlands |
| < 1.7-2.5 mmol/L | Patients with atherosclerosis or CVD | Norwegian Directorate of Health  Norheim et al. (2009)[14] | Norway |
| < 80 mg/dL (2.1 mmol/L) or > 40% reduction | Patients with ischaemic stroke or TIA and CHD, diabetes mellitus, current smoking, metabolic syndrome, or evidence of atherosclerotic origin (high risk); or ischaemic stroke or TIA patients with evidence of unstable atheroma or arterial-arterial embolisms | Chinese Expert Panel  Hankey et al. (2010)[4] | China |
| ≤ 2.0 mmol/L | Adults with clinical evidence of CVD | National Collaborating Centre for Primary Care and Royal College of General Practitioners  Cooper et al. (2008)[15] | UK |

CHD = coronary heart disease; CV = cardiovascular; CVD = cardiovascular disease; EAS = European Atherosclerosis Society; ESC = European Society of Cardiology; FRS = Framingham Risk Score; HDL-C = high-density lipoprotein cholesterol; LDL-C = low-density lipoprotein cholesterol; MI = myocardial infarction; NCEP-ATP-III = National Cholesterol Education Program–Adult Treatment Panel III; NR = not reported; SCORE = Systematic Coronary Risk Evaluation; T1DM = type 1 diabetes mellitus; T2DM = type 2 diabetes mellitus; TIA = transient ischaemic attack; UK = United Kingdom; US = United States.

^a^ CHD includes history of MI, unstable angina, stable angina, coronary artery procedures (angioplasty or bypass surgery), or evidence of clinically significant myocardial ischaemia.

^b^ CHD risk equivalents include clinical manifestations of non-coronary forms of atherosclerotic disease (peripheral arterial disease, abdominal aortic aneurysm, and carotid artery disease [TIAs or stroke of carotid origin or > 50% obstruction of a carotid artery]), diabetes, and 2 or more risk factors with 10-year risk for CHD > 20%.

^c^ Risk factors include low HDL-C < 40 mg/dL, family history of premature coronary artery disease in first-degree relatives (a man aged < 55 years or a woman < 65 years), and impaired glucose tolerance.

^d^ Risk factors include cigarette smoking, hypertension (blood pressure ≥ 140/90 mmHg or on antihypertensive medication), low HDL-C (< 40 mg/dL), family history of premature CHD (CHD in male first-degree relative < 55 years of age; CHD in female first-degree relative < 65 years of age), and age (men ≥ 45 years; women ≥ 55 years).

Table S3. Patients With Pre-existing Conditions Not Achieving Target LDL-C Levels

| Author (Year) | Country | Study Design/Sample Size | Target LDL-C Levels | Patients Who Do Not Achieve the LDL-C Target (%) |
| --- | --- | --- | --- | --- |
| Arafah et al. (2014)[16] | Arabian Gulf countries (Bahrain, Oman, Qatar, UAE, KSA, and Kuwait) | Multicentre, non-interventional survey N = 5,276 (5,457 enrolled) | Primary target LDL-C goals according to the updated NCEP-ATP-III guidelines (2004)  Secondary LDL-C target goals according to the TJETF guidelines and the proportion of patients in primary prevention, secondary prevention and MS achieving both of these target goals | **NCEP-ATP-III:** MS: 1,054/1,945 (54.0) FH: 34/63 (54.0) PAD: 112/149 (75.2) CHD: 66.6% Cerebrovascular disease: 72.4% Hypertension: 49.9% Family history of premature CVD: 51.6% Smoker: 54.1% **TJETF target:** CHD: 29.0% Without CHD: 45.3% Cerebrovascular disease: 38.5 No cerebrovascular disease: 40.4% MS: 45.7% Without MS: 37.2% FH: 61.9% |
| Assmann et al. (2006)[17] | Germany | 4E-Registry study (observational):  n = 52400 patients | Intermediate risk (10-year risk of CHD 10%-20%: 3.4 mmol/L (130 mg/dL)  High risk (10-year risk > 20%): 2.6 mmol/L (100 mg/dL) | **Individual treatment goals for low, intermediate, and high risks at 9 months:** Men without DM = 71.1% Men with DM = 74.7% Women without DM = 55.0% Women with DM = 76.0% **LDL-C < 100 mg/dL at 9 months:** Men without DM = 88.0% Men with DM = 84.1% Women without DM = 90.6% Women with DM = 87.7% |
| Chan et al. (2012)[18] | China (Hong Kong) | Cross-sectional observational study as part of the CEPHEUS Pan-Asian Survey N = 561 | **The updated 2004 NCEP-ATP-III guidelines:**  LDL-C goal of < 100 mg/dL for high risk (CHD or CHD risk equivalents and 10-year risk > 20%), with the option to further lower the goal to < 70 mg/dL for those patients at very high risk, and a LDL-C goal of 130 mg/dL for those patients at moderately high risk (2 or more risk factors and 10-year risk of 10%-20%), with an optional goal of 100 mg/dL | FH: 1/1 (100%) MS: 47/279 (16.8%) No MS: 48/280 (17.1%) MS and low HDL-C: 33/272 (12.1%) MS without low HDL-C: 62/288 (21.5%) High BP (≥ 130 ≥ 85 mmHg): 60/282 (21.3%) No high BP: 36/279 (12.9%) Diabetes: 29/230 (12.6%) No diabetes: 67/331 (20.2%) Hypertension: 64/418 (15.3%) CHD: 89/534 (16.7%) PAD: 5/20 (25.0%) Family history of premature CHD: 17/72 (23.6%) |
| Chong et al. (2011)[19] | Singapore | Prospective cohort study N = 105 | Target LDL-C level for patients with CAD was < 100 mg/dL (< 2.6 mmol/dL) | **Baseline:** < 100 mg/dL: 91/105 (86.7%) < 80 mg/dL: 102/105 (97.2%) **Post-statin monotherapy:** < 100 mg/dL: 85.7% < 80 mg/dL: 102/105 (97.2%) **Post-ezetimibe therapy:** < 100 mg/dL: 36.2% < 80 mg/dL: 60.0% (Significant difference post ezetimibe; *P* < 0.001) |
| Egan et al. (2013)[20] | US | NHANES, a representative sample of the US civilian population N = 82,210 Patients with hypertension and hyperlipidaemia = 4,589 | Cholesterol control was based principally on NCEP-ATP-III goals  Both NCEP-ATP-III and NCEP-ATP-II (1993) goals for LDL-C in high-risk patients were < 100 mg/dL | 2005-2010: In hypertensive patients with LDL-C, 54.6% were uncontrolled (8.1% were treated) Concomitant control of hypertension to between < 140 and < 90 and LDL-C to NCEP-ATP-III targets rose approximately 6-fold from 1988-1994 to 2005-2010 For all hypertensive patients, 21.5% were treated and uncontrolled |
| Elis et al. (2011)[21] | Israel | Cross-sectional database study N = 93,714 CHD = 24,083 DM = 54,261 Both CHD and DM = 15,370 | LDL-C < 100 mg/dL | **LDL-C < 100 mg/dL:** DM: 50% CHD: 43% Both: 33% **LDL-C < 70 mg/dL:** DM: 86%-87% CHD:86%-87% Both: 76% |
| Ferrer-Garcia et al. (2008)[22] | Spain | Observational study of patients with T2DM N = 202 (188 included in analysis) | LDL-C levels of < 2.6 mmol/L | **LDL-C < 2.6 mmol/L:**  Overall: 33.5% (63/188) **LDL-C < 1.82 mmol/L:** Overall: 93.1% (175/188) |
| Foley et al. (2006)[23] | US | Cross-sectional survey of 107 physicians’ attitudes and beliefs about hyperlipidaemia; physicians provided treatment histories for 1,187 patients with CHD or RE | LDL-C < 100 mg/dL | **Follow-up treatment for those not at goal with initial therapy (N = 843):** Pretreatment LDL-C group: 100-129 mg/dL: 51.0%  130-159 mg/dL: 51.3% 160-189 mg/dL: 56.6% > 190 mg/dL: 72.2% LDL-C missing: 57.4% |
| Gajdos et al. (2007)[24] | Slovakia | Observational study N = 5,640 | NCEP-ATP-III | CHD: 88.1% DM: 88.2% CHD + DM: 86.6% |
| Iglseder et al. (2005)[25] | Austria | Observational study N = 9,274 | **NCEP-ATP-III guidelines:**  2+ risk factors: LDL-C < 130 mg/dL  CHD or risk equivalent: LDL-C < 100 mg/dL | **CHD or risk equivalent:** Overall: 2,233/2,381 (93.8%) Treated: 395/443 (89.2%) **Diabetic patients:** Overall: 872/954 (91.4%) Treated: 112/128 (80.5%) |
| Ilerigelen et al. (2007)[26] | Turkey | Open-label, prospective, multi-centre study N = 154 | **NCEP-ATP-III guidelines:**  ≥ 2 risk factors: < 3.37 mmol/L  CHD or risk equivalents: < 2.59 mmol/L | CHD or risk equivalents: 32.6% |
| Jaussi et al. (2010)[27] | Switzerland | Prospective cross-sectional survey Phase 1: N = 23,892  Low risk: n = 11,363 Medium risk: n = 2,914 High risk: n = 9,615 Phase 2 (high-risk patients who did not reach LDL-C goal could be included) High risk: n = 3,250 complete data sets were available for 3,097 (95%) | High risk (PROCAM score > 10% or known CHD or DM): < 2.6 mmol/L Medium risk (PROCAM score between 10% and 20%): < 3.4 mmol/L | **Phase 1:** DM only: 64% (2,470/3,868) CHD only: 55% (1,763/3,185) DM and CHD: 48% **Phase 2:** DM only: 67% (822/1,231) CHD: 66% (598/909) DM and CHD: 66% |
| Krasuski et al. (2005)[28] | US | Group A: N = 966  (841 in analysis population) Retrospective database study Group B: N = 104 prospective study of patients in a lipid clinic | NCEP-ATP-III LDL-C goal attainment | **DM:** Simvastatin: 247/309 (80%) Atorvastatin: 130/309 (42%) **All coronary disease, DM, or PVD:** Simvastatin: 536/662 (81%) Atorvastatin: 271/662 (41%) **All hypertensives:** Simvastatin: 412/564 (73%) Atorvastatin: 203/564 (36%) |
| Krause et al. (2008)[29] | Brazil | Descriptive, transversal, and observational study N = 312 | Patients without CVD: < 130 mg/dL Patients with CVD: < 100 mg/dL | Untreated patients with CVD: 74.2% (target < 100 mg/dL) Treated patients with CVD: 47.4% (target < 100 mg/dL) |
| Li et al. (2010)[30] | US | NHANES; a series of multi-stage surveys of the non-institutionalised civilian population in the US N = 5,098 | **NCEP-ATP-III guidelines:**  Patients with CVD or DM into a high-risk category with the goal of LDL-C < 100 mg/dL (2.6 mmol/L), and patients with both CVD and DM into a very high-risk category with the optional target of LDL-C < 70 mg/dL (1.7 mmol/L) | **Uncontrolled LDL-C:** 1999-2002: Patients with DM: 70.5% Patients with IHD: 71.1% Patients with both: 89.0% 2003-2006: Patients with DM: 60.1% Patients with IHD: 54.6% Patients with both: 83.4% |
| Maki et al. (2006)[31] | US | The analysis compared the percentage of patients reaching target lipid levels according to NCEP and CWG guidelines among participants of the NCEP Evaluation Project Utilising Novel E-technology II survey N = 4,885 | **NCEP-ATP-III risk categories:**  2+ risk factors (≤ 20% 10-year risk): < 3.3 mmol/L CAD or CAD risk equivalents, (> 20% 10-year risk): < 2.58 mmol/L **CWG risk categories:**  Moderate (11%-19% 10-year risk): < 3.5 mmol/L  High (CAD or CAD risk equivalents or ≥ 20% 10-year risk): < 2.5 mmol/L | **NCEP:** CAD + CAD RE: 43.0% **Patients in CAD or CAD risk equivalents or high risk:** NCEP: CAD: 38.0% DM (no CAD): 45.0% Other^a^: 59.6 CWG: CAD: 42.3% DM (no CAD): 49.9% Other^a^: 63.1% |
| Michel et al. (2008)[32] | Luxembourg | Patient survey—the CEPHEUS study  N = 706 | TJETF and 2004 NCEP-ATP-III guidelines | **TJETF:** Patients with DM and without CVD (target < 100 mg/dL; secondary prevention): 61.0% Patients with DM and CVD (very high risk; target LDL-C < 70 mg/dL and TC < 175 mg/dL): 82.5% |
| Mosca et al. (2005)[33] | US | Historical prospective cohort analysis of an integrated, managed-care database of high-risk women with evidence of CVD or RE N = 8,353 | LDL-C < 100 mg/dL | At baseline 17% had an LDL-C < 100 mg/dL; therefore, 83% were above target At 36 months, 29% had an LDL-C < 100 mg/dL; therefore, 71% did not reach targets |
| Munawar et al. (2013)[34] | Indonesia | Prospective, cross-sectional survey on subjects on lipid-lowering pharmacological therapy  Part of the Pan-Asian CEPHEUS study N = 834 (979 enrolled) | **Updated 2004 NCEP-ATP-III guidelines:**  Moderate risk (2+ risk factors, 10-year risk < 10%): < 130 mg/dL High risk (CHD or CHD risk equivalents, 10-year risk > 20%): < 100 mg/dL  Very high risk (established CVD plus 1 or more risk factors): < 70 mg/dL | Diabetes: 77.1% (247/320) CHD: 81.3% (235/289) Carotid artery disease: 90.5% (57/63) PAD: 84.7% (83/98) MS: 72.0% Multiple risk factors (10-year CHD risk > 20%): 77.9% (113/145) |
| Nitiyanant et al. (2008)[35] | Thailand | A multicentre cross-sectional, nationwide survey (LTAP-II) N = 1,921 | NCEP-ATP-III | CHD or CHD equivalents: 751/1,148 (65.4%) |
| Paragh et al. (2007)[36] | Hungary | Multicentre, observational study N = 440 | **Hungarian national guidelines:**  < 2.5 mmol/L (< 100 mg/dL) in the high-risk group  < 3.0 mmol/L (< 117 mg/dL) in the lower-risk group | CHD or CHD risk equivalents: 74.4% Without CHD or risk equivalent: 79.7% |
| Poli et al. (2011)[37] | Italy | Using data from the CHECK study, this work defined the distribution of LDL-C targets and the individual distance from target in a sample of about 5,500 subjects, representative of the Italian adult population N = 5,456 High or very high risk: n = 1,325 | Medium GCVR: 10% ≤ GCVR < 20%: < 130 mg/dL  High GCVR: diagnosed coronary disease or equivalent (diabetes, CVD, OALL) or GCVR ≥ 20%: < 100 mg/dL  Very high diagnosed coronary disease plus diabetes, or smoking, or MS, or uncontrolled hypertension: < 70 mg/dL | **1/3 of the CHECK cohort:** High or very high risk: 4419/5456 (81%) Non-statin treated: 84%  Statin treated: 73% |
| Save et al. (2006)[38] | Unclear: possibly India | Observational study and clinical trial of patients with DM N = 110 | < 100 mg/dL | **N = 103:** Week 24: overall, 12.9% Week 12: overall, 63.0% During the next 12 weeks: 45.9% LDL-C < 70%: 86.4% |
| Wang et al. (2014)[39] | Taiwan | Prospective, cross-sectional survey N = 999 | **NCEP-ATP-III update 2004 targets:**  CHD or CHD risk equivalents < 100 mg/dL (optional < 70 mg/dL)  2+ risk factors, 10-year risk: > 20%: < 100 mg/dL  2+ risk factors, 10-year risk: 10%-20%: < 130 mg/dL (optional < 100 mg/dL)  2+ risk factors, 10-year risk: < 10%: < 130 mg/dL  High risk: < 100 mg/dL  Very high risk: < 70 mg/dL | Diabetes: 54% (from graph) Multiple risk factors other than CHD or equivalent: 63% Without multiple risk factors: 47% MS: 57% Without MS: 34% |
| Wong et al. (2006)[40] | US | NHANES 2001-2002 N = 2,864 | LDL-C ≤ 130 mg/dL (≤ 100 mg/dL in patients with DM or CVD) | 89.0% of patients with hypercholesterolaemia were uncontrolled (91.0% for patients with both conditions) Patients without MS, DM, or CVD: 94.5% had controlled hypertension and hypercholesterolaemia Uncontrolled hypertension and hypercholesterolaemia: Patients with MS: 90.6% Patients with DM: 98.0% Patients with CVD: 85.0% Patients with CVD + DM or MS: 84.0% |
| Wong et al. (2013)[41] | US | NHANES N = 2,509 Patients with hyperlipidaemia = 1,129 | Target levels for LDL-C were adapted from the NCEP but updated on the basis of more recent recommendations for an optional LDL-C goal of < 70 mg/dL for those patients with pre-existing CHD | Treated patients with CHD: 74.2% did not achieve LDL-C target < 70 mg/dL; 32.5% did not achieve LDL-C target < 100 mg/dL Patients on treatment not achieving goals (< 70 mg/dL or < 100 mg/dL if CHD): Disease group: CVD: 65.3% CHD: 74.2% Heart failure: 58.4% Stroke: 58.3% Diabetes: 37.9% MS: 42.1% Chronic kidney disease: 47.6% |
| Yiginer et al. (2010)[42] | Turkey | Cross-sectional n = 194 patients | High risk (per NCEP-ATP-III guidelines): 100 mg/dL | Fewer primary prevention patients with DM achieved target levels than secondary prevention patients with DM |

BMI = body mass index; BP = blood pressure; CAD = coronary artery disease; CHD = coronary disease; CVD = cardiovascular disease; CWG = Canadian Working Group; DM = diabetes mellitus; FH = familial hypercholesterolaemia; HDL-C = high-density lipoprotein cholesterol; IHD = ischaemic heart disease; KSA = Kingdom of Saudi Arabia; LDL-C = low-density lipoprotein cholesterol; LTAP-II = Lipid Treatment Assessment Project II in Thailand; MS = metabolic syndrome; NCEP = National Cholesterol Eduction Program; NCEP-ATP-II = National Cholesterol Eduction Program–Adult Treatment Panel II; NCEP-ATP-III = National Cholesterol Eduction Program–Adult Treatment Panel III; NHANES = National Health and Nutrition Examination Survey; OALL = Obliterating atherosclerosis of lower limbs; PAD = peripheral artery disease; PROCAM = Prospective Cardiovascular Münster study; PVD = peripheral vascular disease; RE = risk equivalent; T2DM = type 2 diabetes mellitus; TC = total cholesterol; TJETF = Third Joint European Task Force; UAE = United Arab Emirates; US = United States.

^a^ Other risk categories include age, gender, race, BMI, blood pressure.

REFERENCES

1. Wascher TC, Paulweber B, Toplak H, Saely C, Drexel H. [Lipids: diagnosis and therapy in type 2 diabetes]. Wien Klin Wochenschr 2012; 124(suppl 2): 28-30.
2. Reiner Z, Catapano AL, Backer GD, Graham I, Taskinen MR, Wiklund O. ESC/EAS guidelines for the management of dyslipidaemias. The Task Force for the management of dyslipidaemias of the European Society of Cardiology (ESC) and the European Atherosclerosis Society (EAS). Eur Heart J 2011; 32: 1769-818.
3. Grundy SM, Cleeman JI, Merz CNB, Brewer HB, Clark LT, Hunninghake DB; for the Coordinating Committee of the National Cholesterol Education Program. Implications of recent clinical trials for the National Cholesterol Education Program Adult Treatment Panel III Guidelines. Circulation 2004; 110: 227-39.
4. Hankey GJ, Wong KSL, Chankrachang S, Chen C, Crimmins D, Frayne J, et al. Management of cholesterol to reduce the burden of stroke in Asia: consensus statement. Int J Stroke 2010; 5: 209-16.
5. Teramoto T, Sasaki J, Ishibashi S, Birou S, Daida H, Dohi S, et al.; Japan Atherosclerosis Society. Executive summary of the Japan Atherosclerosis Society (JAS) guidelines for the diagnosis and prevention of atherosclerotic cardiovascular diseases in Japan—2012 version. J Atheroscler Thromb 2013; 20(6): 517-23.
6. Austrian Lipid Consensus 2010. Management of dyslipidemia in the prevention of vascular complications. Joint Consensus Statement of Austrian eight professional societies. <http://www.oedg.org/pdf/Lipidkonsensus_2010.pdf> (20 May 2014, date last accessed).
7. Huber K, Saely CH, Drexel H, Francesconi M, Gaul G, Glehr R, et al. Practical implementation of the guidelines for LDL-lowering. J Cardiol 2011; 18(3-4): 96-9.
8. Rodondi N, Gencer B, Collet TH, Battegay E. Ab welchem Cholesterinwert soll in der Schweiz eine Behandlung erfolgen? Schweiz Med Forum 2011; 11(27): 467-72.
9. South African Medical Association and Lipid and Atherosclerosis Society of Southern Africa Working Group. Diagnosis, management and prevention of the common dyslipidaemias in South Africa—clinical guideline, 2000. SA Med J 2000; 90: 164-78.
10. Butler N. National guidelines at a glance: hypercholesterolaemia. Prof Nursing Today 2010; 14(5): 26-31.
11. Anderson TJ, Gregoire J, Hegele RA, Couture P, Mancini GBJ, McPherson R, et al. 2012 update of the Canadian Cardiovascular Society guidelines for the diagnosis and treatment of dyslipidemia for the prevention of cardiovascular disease in the adult. Can J Cardiol 2013; 29: 151-67.
12. Chung EE. Best practice guidelines for treatment of hypercholesterolaemia: a statement from the Caribbean Cardiac Society. West Indian Med J 2008; 57(1): 48-53.
13. Smulders YM, Burgers JS, Scheltens T, van Hout BA, Wiersma T, Simoons ML. Clinical practice guideline for cardiovascular risk management in the Netherlands. Neth J Med. 2008;66(4):169-74.
14. Norheim OF, Gjelsvik B, Kjeldsen SE, Klemsdal TO, Madsen S, Meland E, et al. Directorate of Health. Guidelines for individual primary prevention of cardiovascular disease. 2009. <http://www.helsedirektoratet.no/publikasjoner/nasjonal-faglig-retningslinje-for-individuell-primerforebygging-av-hjerte-og-karsykdommer/Publikasjoner/IS-1550.pdf> (12 May 2014, date last accessed).
15. Cooper A, Nherera L, Calvert N, O’Flynn N, Turnbull N, Robson J, et al. Clinical guidelines and evidence review for lipid modification: cardiovascular risk assessment and the primary and secondary prevention of cardiovascular disease. London: National Collaborating Centre for Primary Care and Royal College of General Practitioners 2008 (revised March 2010): 1-236.
16. Arafah M, Al-Hinai AT, Mahmeed WA, Al-Rasadi K, Al Tamimi O, Al Herz S, et al. Centralized pan-Middle East Survey on the undertreatment of hypercholesterolemia: results from the CEPHEUS study in Arabian Gulf countries. Angiology 2014 Nov; 65(10): 919-26. doi: 10.1177/0003319713512414. Epub 2013 Dec 3.
17. Assmann G, Benecke H, Neiss A, Cullen P, Schulte H, Bestehorn K. Gap between guidelines and practice: attainment of treatment targets in patients with primary hypercholesterolemia starting statin therapy. Results of the 4E-Registry (Efficacy Calculation and Measurement of Cardiovascular and Cerebrovascular Events Including Physicians’ Experience and Evaluation). Eur J Cardiovasc Prev Rehabil 2006; 13(5): 776-83.
18. Chan RH, Chan PH, Chan KK, Lam SCC, Hai JJ, Wong MKL, et al. The CEPHEUS Pan-Asian survey: high low-density lipoprotein cholesterol goal attainment rate among hypercholesterolaemic patients undergoing lipid-lowering treatment in a Hong Kong regional centre. Hong Kong Med J 2012; 18(5): 395-406.
19. Chong E, Shen L, Poh KK. Half-dose ezetimibe add-on to statin therapy is effective in improving resistant hyperlipidaemia in Asian patients with ischaemic heart disease. Singapore Med J 2011; 52(6): 400-4.
20. Egan BM, Li J, Qanungo S, Wolfman TE. Blood pressure and cholesterol control in hypertensive hypercholesterolemic patients: national health and nutrition examination surveys 1988-2010. Circulation 2013; 128(1): 29-41.
21. Elis A, Chodick G, Heymann AD, Kokia E, Flash S, Lishner M, et al. The achievement of target cholesterol level differs between coronary heart disease and diabetic patients. Eur J Intern Med 2011;22(3):262-5.
22. Ferrer-Garcia JC, Sanchez-Ballester E, Albalat-Galera R, Berzosa-Sanchez M, Herrera-Ballester A. Efficacy of atorvastatin for achieving cholesterol targets after LDL-cholesterol based dose selection in patients with type 2 diabetes. J Cardiovasc Pharmacol Ther. 2008; 13(3): 183-8.
23. Foley KA, Denke MA, Kamal-Bahl S, Simpson R Jr, Berra K, Sajjan S, et al. The impact of physician attitudes and beliefs on treatment decisions: lipid therapy in high-risk patients. Med Care 2006; 44(5): 421-8.
24. Gajdos M, Krivosikova Z, Uhliar R. A critical gap between recommended and achieved LDL-cholesterol levels. Results of statin therapy in Slovakia. Bratisl Lek Listy 2007; 108(9): 388-92.
25. Iglseder B, Moroder T, Staffen W, Ladurner G. High prevalence and undertreatment of hypercholesterolaemia in participants in a public stroke prevention programme in Austria. Clin Drug Investig 2005; 25(11): 709-17.
26. Ilerigelen B, Uresin Y, San M, Kültürsay H, Güneri S, Serdar OA, et al. Efficacy and safety of extended-release fluvastatin in Turkish patients with hypercholesterolaemia: TULIPS (Turkish Lipid Study). Curr Med Res Opin 2007; 23(5): 1093-102.
27. Jaussi A, Noll G, Meier B, Darioli R. Current cardiovascular risk management patterns with special focus on lipid lowering in daily practice in Switzerland. Eur J Cardiovasc Prev Rehabil 2010; 17(3): 363-72.
28. Krasuski RA, Doeppenschmidt D, Henry JS, Smith PB, Adinaro J, Beck R, et al. Conversion to atorvastatin in patients intolerant or refractory to simvastatin therapy: the CAPISH study. Mayo Clin Proc 2005; 80(9): 1163-8.
29. Krause MP, Hallage T, Miculis CP, Gama MP, Silva SG. Analysis of the lipid profile of elderly women in Curitiba – Parana. Arq Bras Cardiol 2008; 90(5): 299-304.
30. Li M, Ong KL, Tse HF, Cheung BM. Utilization of lipid lowering medications among adults in the United States 1999-2006. Atherosclerosis 2010;208(2):456-460.
31. Maki KC, Davidson MH, Dicklin MR. A comparison of Canadian and American guidelines for lipid management using data from the National Cholesterol Education Program Evaluation Project Utilizing Novel E-technology (NEPTUNE) II Survey. Can J Cardiol 2006; 22(4): 315-22.
32. Michel G, Muller P. Pan-European survey on the undertreatment of hypercholesterolemia in hypolipidemias (Luxembourg). Bull Soc Sci Med Grand Duche Luxemb 2008; (4): 509-16.
33. Mosca L, Merz NB, Blumenthal RS, Cziraky MJ, Fabunmi RP, Sarawate C, et al. Opportunity for intervention to achieve American Heart Association guidelines for optimal lipid levels in high-risk women in a managed care setting. Circulation 2005; 111(4): 488-93.
34. Munawar M, Hartono B, Rifqi S. LDL cholesterol goal attainment in hypercholesterolemia: CEPHEUS Indonesian survey. Acta Cardiologica Sinica 2013; 29(1): 71-81.
35. Nitiyanant W, Sritara P, Deerochanawong C, Ngarmukos P, Koanantakul B. Lipid treatment assessment project II in Thailand (LTAP-II Thailand). J Med Assoc Thai 2008; 91(6): 836-45.
36. Paragh G, Mark L, Zamolyi K, Pados G, Ofner P. Lipid-modifying therapy and attainment of cholesterol goals in Hungary: the return on expenditure achieved for lipid therapy (REALITY) study. Clin Drug Investig 2007; 27(9): 647-60.
37. Poli A, Casula M, Tragni E, Brignoli O, Filippi A, Cricelli C, et al. Reaching LDL-C targets in high-risk patients requires high-efficacy cholesterol-lowering drugs in more than 50% of cases. The results of the CHECK study. Pharmacol Res. 2011 Oct; 64(4): 393-6.
38. Save V, Patil N, Moulik N, Rajadhyaksha G. Effect of atorvastatin on type 2 diabetic dyslipidemia. J Cardiovasc Pharmacol Ther 2006; 11(4): 262-70.
39. Wang KF, Chang CC, Wang KL, Wu CH, Chen LC, Lu TM, et al. Determinants of low-density lipoprotein cholesterol goal attainment: insights from the CEPHEUS Pan-Asian Survey. J Chin Med Assoc 2014 Feb; 77(2): 61-7.
40. Wong ND, Lopez V, Tang S, Williams GR. Prevalence, treatment, and control of combined hypertension and hypercholesterolemia in the United States. Am J Cardiol 2006; 98(2): 204-8.
41. Wong ND, Chuang J, Wong K, Pham A, Neff D, Marrett E. Residual dyslipidemia among United States adults treated with lipid modifying therapy (data from National Health and Nutrition Examination Survey 2009-2010). Am J Cardiol 2013;112(3):373-379.
42. Yiginer O, Ozmen N, Ozcelik F, Inanç T, Kardeşoğlu E, Uz O, et al. Adherence to statin therapy and LDL cholesterol goal attainment in type 2 diabetics and secondary prevention patients: the role of education and knowledge. Turk Kardiyol Dern Ars 2010; 38(8): 544-50.
